# The Genetics and the Genomics of Primary Congenital Glaucoma

**DOI:** 10.1155/2015/321291

**Published:** 2015-09-16

**Authors:** Raffaella Cascella, Claudia Strafella, Chiara Germani, Giuseppe Novelli, Federico Ricci, Stefania Zampatti, Emiliano Giardina

**Affiliations:** ^1^Department of Biomedicine and Prevention, School of Medicine, University of Rome “Tor Vergata”, 00133 Rome, Italy; ^2^Emotest Laboratory, 80078 Pozzuoli, Italy; ^3^Molecular Genetics Laboratory UILDM, Santa Lucia Foundation, 00142 Rome, Italy; ^4^UOSD Retinal Pathology PTV Foundation “Policlinico Tor Vergata”, 00133 Rome, Italy; ^5^Neuromed IRCCS, 86077 Pozzilli, Italy

## Abstract

The sight is one of the five senses allowing an autonomous and high-quality life, so that alterations of any ocular component may result in several clinical phenotypes (from conjunctivitis to severe vision loss and irreversible blindness). Most parts of clinical phenotypes have been significantly associated with mutations in genes regulating the normal formation and maturation of the anterior segments of the eye. Among the eye anterior segment disorders, special attention is given to Glaucoma as it represents one of the major causes of bilateral blindness in the world, with an onset due to Mendelian or multifactorial genetic-causative traits. This review will point out the attention on the Primary Congenital Glaucoma (PCG), which is usually transmitted according to an autosomal-recessive inheritance pattern. Taking into consideration the genetic component of the PCG, it is possible to observe a strong heterogeneity concerning the disease-associated loci (*GLC3*), penetrance defects, and expressivity of the disease. Given the strong PGC heterogeneity, pre- and posttest genetic counseling plays an essential role in the achievement of an appropriate management of PCG, in terms of medical, social, and psychological impact of the disease.

## 1. Introduction

The sight is one of the five senses allowing an autonomous and high-quality life. Hence, sight defects may turn out to be really restricting for the life quality, representing a discriminating or even a life-threatening issue, especially in developing countries. As a matter of fact, about the 75% of information we receive about the world around us relies on the ability of the eyes to receive the light and transform it into a “picture” [1].

Given the critical importance of the sight organ, it is not surprising that alterations or malformations of any ocular component may result in several clinical phenotypes, ranging from conjunctivitis to Cataract until the severe vision loss and the irreversible blindness. Ocular clinical syndromes may develop owing to environmental agents (infections acquired during pregnancy or later in the life, aging processes, and systemic disorders), which can affect the ocular structures at the developmental or mature stages. However, most parts of clinical phenotypes have been significantly associated with mutations in genes regulating the normal formation and maturation of both posterior and anterior segments of the eye [2]. This review will be particularly focused on the genetics and genomics of the anterior segment disorders.

## 2. Genetic and Phenotypic Overview on the Eye Anterior Segment Mendelian Disorders

The clinical manifestations characterizing the anterior segment disorders reflect both phenotypic and genotypic heterogeneity, with multiple overlapping symptoms and associated genes [3]. Firstly, it is important to distinguish the anterior segment disorders characterized by a Mendelian or simple genetics from the disorders developed in consequence of complex or multifactorial traits. In particular, while the Mendelian diseases are due to causative rare mutations in specific genes (causative gene) and are inherited according to Mendel's laws, multifactorial diseases are caused by an interplay between several genetic and environmental triggering factors [2, 4]. In the large majority of cases, the genetic variants, associated with multifactorial diseases, are not rare mutations but rather polymorphisms, located on susceptibility genes and reported at high frequencies in normal/nonaffected population. These genetic markers are not sufficient to cause the disease but they only represent risk factors, determining a higher susceptibility to a particular disease. As mentioned below, it is interesting to notice that the presence of modifier genes, polymorphisms, or mutations may also be responsible for the variable expressivity occurring in Mendelian and complex diseases [5, 6]. In addition, the recognition of the distinctive features related to each anterior segment disorder can be complicated by different factors [5] such as

- the presence of extraocular (systemic) clinical manifestations, as facial dysmorphisms, dental dysplasia, redundant preumbilical skin, and short stature;
- heterogeneous inheritance patterns (mainly autosomal-dominant and/or autosomal-recessive inheritance patterns);
- developmental abnormalities occurring during embryogenesis and maturation of the eye anterior segment tissues (leading to congenital diseases);
- foreign events, such as inflammations, infections, and trauma.

Among the most studied Mendelian diseases, it is important to mention Axenfeld-Rieger syndrome (ARS, #180500, #601499, and #602482), a rare disorder which affects 1 : 200.000 individuals and follows an autosomal-dominant inheritance pattern. The ARS etiopathology is characterized by an abnormal development of the anterior chamber, the cornea, and the iris. Clinical hallmarks of the disease include hypoplasia, corectopia, polycoria, posterior embryotoxon, and peripheral anterior synechiae (iris strands). Facial, dental, umbilical, and skeletal defects are also usually present as extraocular manifestations [5, 7].

Another Mendelian disorder affecting the anterior segment of the eye is referred to as Peters' Anomaly (#604229). It presents a heterogeneous inheritance, mostly autosomal-recessive, although autosomal-dominant and sporadic cases have been reported. The disease is caused by an incomplete separation of the cornea from the iris or the lenses, resulting in a variable central corneal opacity among patients. Systemic abnormalities reported in patients with Peters' Anomaly are short stature, cleft lip palate, growth, and mental delay [5, 8].

The complete or partial absence of the iris results in a pathological phenotype known as Aniridia (#106210). It affects 1 : 50.000–100.000 newborns worldwide and it is inherited in autosomal-dominant pattern. Aniridia is characterized by a reduced visual acuity and photophobia. Suddenly, it is associated with behavioral problems, developmental delay, and difficulties in detecting the odors [5, 9].

Concerning the disease phenotypes associated with malformations of the sclera and the cornea, it is important to mention the Sclerocornea and the Megalocornea. Sclerocornea (#18170) is a congenital disorder characterized by the absence of separation between sclera and cornea, which presents, as a consequence, a flat curvature. The malformation results in a nonprogressive corneal opacification (peripheral, sectoral, or central) [5, 10]. Megalocornea (#309300) instead is generally inherited in an X-linked recessive pattern and it consists of an enlargement of the corneal diameter (>13 mm) at birth, a deep anterior chamber, and normal IOP [5, 11].

Among the anterior segment disorders, particular attention has been paid to Keratoconus and Glaucoma, since both of the diseases are characterized by the presence of both Mendelian and multifactorial traits which give rise to different forms of Keratoconus and Glaucoma.

Keratoconus (#148300) is a noninflammatory degenerative disorder of the cornea affecting 1 in 2.000 individuals. The disease is due to the bulging and distortion of the corneal curvature and surface, which lead to the loss of visual acuity. A number of environmental factors have been associated with the development of Keratoconus, especially long-term contact lens wear, chronic eye rubbing, and atopy of the eye. However, the observation of familiarity transmission and studies on monozygotic and dizygotic twins have demonstrated that Keratoconus is characterized by a significant genetic component. In fact, the disease showed to be mostly transmitted in an autosomal-dominant inheritance pattern, although reduced penetrance and autosomal-recessive transmission have been reported [12].

Concerning Glaucoma several types of diseases can be distinguished, depending on the presence of causative Mendelian or multifactorial characters. In multifactorial Glaucoma, the clinical phenotype originates from the interaction between genetic/molecular (*LOXL1* genetic polymorphisms/retinal ganglionic cell death) and environmental (aging, diet, stress, and life-style) factors [13–15]. Although the hereditary component of the disease remains poorly understood, it is known that it can affect both genders, with higher risk for male subjects, advanced age people, and African descendants [16]. This form of Glaucoma is quite similar to several multifactorial, chronic, and age-related degenerative diseases, such as Osteoporosis, Osteoarthritis, Alzheimer's Disease, Coronary Artery Disease, Cataract [17]. In fact, multifactorial Glaucoma is a chronic neurodegenerative process, with an onset in the middle age and a slow progressive course, without particular signs or symptoms. Subjects affected by Glaucoma show a reduction of the visual field, blurry vision, decreased sensitivity to color, and contrast [16]. Over the multifactorial form, it is important to remark the existence of Glaucomas which are characterized by Mendelian inheritance (mainly autosomal-recessive and autosomal-dominant). Further details about clinical and genetic profile of Mendelian disease will be hereafter given.

Over the phenotypic manifestations of the anterior segment diseases, it is important to take into consideration the genetic background of these disorders. In fact, traditional linkage and positional cloning approaches identified a number of “modifier genes” associated with the development, inheritance pattern, variable expressivity, and phenotype of the anterior segment disorders. Such genetic modifiers consist of mutations or polymorphisms in specific genes, involved in the pathological mechanisms responsible for the disease phenotype [5, 13]. However, the identification of disease-causative genes led to the observation that not only is more than one gene mutation accountable for the same clinical condition, but also variable expressivity and incomplete penetrance can occur in patients carrying the same mutation. In addition, Genome Wide Association Studies (GWAS) allowed the identification of other genetic modifiers, especially common genetic variants associated with the individual susceptibility to eye anterior segment disorders. Each disease-associated individual variant confers a low risk when taken alone, while a set of low-risk variants provide a higher predictive value of the overall susceptibility to the disease [5]. The genes involved in the pathogenesis of these disorders include transcription factors in the majority of cases, although some genes code for transporters and glycosylation proteins. Given their function, mutations in the disease-causative genes will obviously have an impact on the downstream gene regulation, the integrity of the mature protein, and its final interaction with the cellular compartments [18]. The common low-risk variants instead are mostly located in noncoding regions (>80%) near the causative genes and essentially affect the overall gene expression patterns (regulation of RNA splicing and transcription activities) [5]. The variability of the gene expression profile can also be altered by epigenetic events (DNA methylation, histone acetylation/deacetylation, and structural chromatin modification), which act on the DNA transcription rather than on the primary DNA sequence [19]. In particular, DNA methyl transferases (DNMTs) were found to be differentially expressed in tissues and related cells of the eye anterior segment, such as the cornea, conjunctiva, anterior lenses, and trabecular meshwork [19]. Altered methylation profiles have been associated with few anterior segment disorders (such as Pterygium, #17800) [20]. Moreover, experiments on human cells and animal models demonstrated that histone modifications (HDA3) and miRNAs (miR29, miR204, miR146a, and miR24) play a role in the development of anterior segment diseases (as the Primary Open Angle Glaucoma, #137760) [21]. However, further research may help to study and discover epigenetic pathways concerning the etiopathogenesis of the congenital disorders affecting the eye anterior segment.

Independently of the phenotype/genotype features of the disorders, it is important to underline that a pathological phenotype does not result from the disruptive effect of one single gene mutation, but from the alteration of the network of genes to which the mutated gene specifically belongs. As a matter of fact, the high phenotypic and genotypic heterogeneity of the anterior segment disorders of the eye is actually the final outcome of the cross-talking between the disease-causative genes and the individual surrounding environment [5].

### 2.1. The Genes Causative of the Eye Anterior Segment Disorders

To date, a number of causative genes have been involved in the pathogenesis of the eye anterior segment disorders, some of which will be briefly described in the following paragraphs.

Both genes* PITX2* and* FOXC1*, located at 4q25 and at 6p25, respectively, code for two transcription factors (Pituitary Homeobox 2 and Forkhead Box C1). Linkage analysis has discovered that specific types of mutations in these genes are associated with specific pathological phenotype. In fact, intragenic mutations and/or deletions in* PITX2* and* FOXC1 *have been reported in ARS syndrome with or without systemic anomalies. Copy Number Variation (CNV) has been found in ARS and Peters' Anomaly, while missense mutations have been identified in patients affected by Peters' Anomaly [3, 22].

*PAX6* was the first gene to be associated with human anterior segment disorder. The gene is mapped on 11p13 and encodes for the transcription factor Paired Box  6. The mutational spectrum of the gene includes nonsense, splicing, insertion, and deletion mutations, resulting in the Aniridia disease. However, some missense mutations in* PAX6* have been reported in Peters' Anomaly and other ocular defects [18, 22].

*B3GALTL* is located on 13q12.3 and is responsible for the beta-1,3-glucosyltransferase (B3Glc-T) enzyme production, which is involved in the glycosylation process of proteins (posttranslational addition of sugar molecules). Deletion and splicing mutations have been reported in patients with Peter Plus Syndrome (PPS, #261540, Peters' Anomaly with variable systemic anomalies) [5, 22].

*SOX2* is mapped on 3q26.3-q27 and codes for the transcription factor SRY-like box  2. It is especially involved in the development of the eyes, so that* SOX2* mutations have been associated with Sclerocornea and other ocular diseases [18].

*CHRDL1* is located on Xq23 and encodes for the ventroptin protein, which works as bone morphogenic protein antagonist. Genomic techniques have associated mutation in* CHRDL1* with the X-linked form of Megalocornea [18].

*VSX1* is situated on 20p11.21 and produces the transcription factor Visual System Homeobox 1. It is functionally involved in the regulation of the events occurring during craniofacial and ocular development. Missense mutations were found to be involved in the pathogenesis of Keratoconus, particularly concerning the autosomal-dominant form with variable expressivity and incomplete penetrance [12].

Other genes related to the above-mentioned and other eye anterior segment disorders (not described in this review) include* FOXE3*,* BMP4*,* BMP7*,* LAMB2*,* COL4A1*,* FGFR2*,* CYP1B1*,* LTBP2*, and* MYOC* [22].

In particular, this review will be focused on the genetic components underlining the congenital form of Glaucoma, a rare pathological condition affecting the anterior chamber drainage structures of the eye.

## 3. Focus on a Specific Disorder of the Anterior Segment of the Eye: The Glaucoma

As mentioned above, in the context of the eye anterior segment disorders special attention is given to Glaucoma. It represents one of the major causes of bilateral blindness in the world and several types of diseases have been classified to date, according to presence of Mendelian or multifactorial genetic-causative traits. In particular, Glaucoma consists of a pathological condition characterized by the progressive death of Retinal Ganglion Cells (RGCs), degeneration of the optical nerve, and vision loss as final result. The real explanation for RGCs death is not yet clear, although the level of IOP is known to play a fundamental role [13]. In particular, the IOP depends on the homeostasis between the aqueous humor produced in the ciliary body (and secreted in the posterior chamber) and its drainage through the trabecular meshwork in the anterior chamber angle. The balanced production and outflow of aqueous humor are constant and generate a positive pressure within the eye (~15 mmHg). Malfunctioning and/or malformations of the trabecular meshwork may result in the elevation of IOP over the limit ranges (>21 mmHg) and consequently the development of Glaucoma. Trabecular meshwork disruption can depend on a number of factors, such as mechanical and oxidative stress, aging, and genetic mutations [23]. The improper drainage of aqueous humor determines the increase of IOP, which in turn generates a mechanical stress and strain on the lamina cribrosa (the exit points of the optic nerve fibers in the sclera). The resulting stress blocks the transport of neurotrophic factors to the RGCs that finally die by apoptosis [24].

Glaucoma can be classified into primary, secondary, and primary congenital. The primary form is a nonsyndromic condition and it does not originate from any previous anterior segment abnormality, trauma, or inflammation. According to the compromised regions, two types of primary Glaucoma can be distinguished: Primary Closed-Angle Glaucoma and Primary Open-Angle Glaucoma. In the first condition, the drainage of aqueous humor is clogged by the closure of the angle between the iris and the cornea, while in the second form the fluid meets high resistance because of a malfunctioning of the trabecular meshwork [23].

The secondary Glaucoma instead is linked to the presence of ocular injuries or systemic conditions (for example, diabetes and long-term corticosteroid use) [23]. Even in this case, Closed-Angle and Open-Angle subtypes can be distinguished. On this subject, the Pseudoexfoliation Syndrome (PEXS) represents one of the major causes of secondary Open-Angle Glaucoma (accounting for the 25% of all Open-Angle Glaucomas). PEXS is described as an age-related systemic disease, which leads to the development of the secondary Glaucoma because of the accumulation of exfoliation material in the trabecular meshwork and consequently the increase of IOP [14].

This review will point out the attention on the Primary Congenital Glaucoma (PCG) which is a rare form representing the 1–5% of all cases of Glaucoma [25]. PCG generally presents an autosomal-recessive inheritance pattern, so that it affects only the 25% of newborns from parents carrying PCG-associated mutations. However, the incidence of PCG is highly heterogeneous in relation to the population, the geographic region, and the prevalence of consanguineous relationships. In fact, the incidence rate is estimated to be 1 : 2.500 in Saudi Arabia and Slovakia Gypsy populations (because of highly frequent consanguineous relations), in contrast with the lower incidence rate recorded among Western populations ranging from 1 : 18.500 and 1 : 30.000 [25, 26].

On the basis of the age of onset, it is possible to classify three subtypes of PCG: neonatal or newborn when presented at the birth or within the 1st month of life; infantile, diagnosed from 1st month to 2 years; and late-onset, recognized after 2 years. Usually, in 70–80% of cases, PCG affect both of the eyes (bilateral form) [27].

The clinical hallmarks of PCG diagnosis include IOP > 21 mmHg, optic cupping, epiphora, corneal edema and Haab striae, globe enlargement (buphthalmos), photophobia, blepharospasm, and hyperlacrimation. The PCG phenotype is due to a trabeculodysgenesis phenomenon, that is, an abnormal development of anterior chamber leading to the enlargement of the trabecular meshwork bundles and consequently reduction of the trabecular area available for the aqueous humor outflow [28]. In addition, the humor drainage is reduced because of the interference of immature iris, ciliary body, and anterior chamber angle structure which appear superimposed on the trabecular meshwork and compromise the aqueous humor outflow. The final result of these anterior segment structural defects and the altered aqueous humor drainage is the elevation of IOP and the enlargement of the entire ocular globe [25].

## 4. Genetics of PCG

Taking into consideration the genetic component of the PCG, it is possible to observe a strong heterogeneity in terms of disease-associated loci, penetrance defects, and expressivity of the disease among the different populations. Concerning the disease-associated loci, the Human Genome Organization established a specific nomenclature for Glaucoma-associated genetic loci. In fact, “GLC” stands for the general name of genes involved in Glaucoma; “1, 2, and 3” indicate the type of primary Glaucoma (Open-Angle, Closed-Angle, and congenital/infantile Glaucoma, resp.); “A, B, C, and D” refer to the progressive genes mapped for each Glaucoma type [26]. This review will focus particularly on* GLC3* loci, since these refer to the primary congenital Glaucoma.

### 4.1. *GLC3* Loci

To date four PCG loci have been classified under* GLC3* subgroup, namely,* GLC3A*,* GLC3B*,* GLC3C*, and* GLC3D*.

The first locus to be associated with PCG was* GLC3A*, mapped on 2p21 chromosomal region. At this locus, about 147 mutations have been found in the gene* CYP1B1*, coding for the homonymous protein (cytochrome P450, family 1, subfamily B, and polypeptide 1) [29, 30].* CYP1B1* consists of 3 exons and 2 introns, of which the first exon is a noncoding region, while the second and third exons are responsible for the production of CYP1B1 protein [25]. The* CYP1B1* mutations can be missense, nonsense, insertions, and deletions and result in the disruption of enzymatic activity and functionality of CYP1B1 protein [25, 31].

The mutations showed an autosomal-recessive inheritance pattern and are the most common variations identified among PCG patients, especially in consanguineous cohorts. However, a high variable distribution of* CYP1B1* mutations has been reported among the different worldwide populations, with 90–100% found in Saudi Arabia and Slovakia Gypsy populations, 14–30% among USA and European populations, and 15–20% reported in Japanese and Chinese populations [31–33]. Interestingly, in families carrying* CYP1B1* mutations cases of incomplete penetrance and variable expressivity have been observed. In fact, patients harboring the same mutations showed a different degree of disease severity, age of onset, or even lack of the disease phenotype [25, 26].

The pathogenetic role of the CYP1B1 protein in PCG has to be yet clarified, although a possible involvement in the metabolic pathways required for eye anterior chamber development is suggested, particularly for the trabecular meshwork formation [26]. On this subject, a recent study conducted on* Cyp1b1* ^−*/*−^ mice demonstrated that* Cyp1b1* deficiency was responsible for the increased oxidative stress and ultrastructural defects in the TM tissue of early-life animals. In particular, the absence of CYP1B1 protein (due to* Cyp1b1* silencing) hampers the proper removal of Reactive Oxygen Species (ROS) and, consequently, compromises the development and differentiation of TM tissue. In addition, the increased oxidative stress in* Cyp1b1* ^−*/*−^ mice caused the decrease of the Periostin (Postn) production, which results in the loss of the mechanical strength and structural integrity of the TM tissue [34].

*GLC3B* and* GLC3C *loci have been associated with the PCG, although no gene has been identified in both regions to date. In particular,* GLC3B *is located at 1p36.2–1p36.1 while* GLC3C* maps on 14q24.3–14q31.1 [25, 26].

Concerning the* GLC3D* locus (14q24), autosomal-recessive null mutations have been reported at this position and located in the* LTBP2* gene.* LTBP2* codes for the Latent Transforming beta Binding Protein 2, a matrix protein playing a role in tissue repair processes and cell adhesion. The role of the PCG still remains to be explained. However, it has been demonstrated that* LTBP2* is expressed in the trabecular meshwork and ciliary body and PCG-causative null mutations have been found in consanguineous Pakistani, European Gypsy, and Iranian families [26, 35].

### 4.2. Other PCG-Associated Genes

Families affected by PCG carried mutations in* MYOC* gene, independently of the presence or absence of* CYP1B1* mutations.* MYOC* is a 3-exon gene, located at 1q24.3–1q25.2 chromosomic region, and codes for the glycoprotein myocilin. The function of the protein is unknown, while its expression has been reported at high level in the trabecular meshwork and the ciliary body. Mutations in* MYOC* have been found to be a leading cause of aqueous outflow obstruction through the trabecular meshwork and of the consequent IOP elevation. A number of* MYOC* mutations have been associated with the juvenile- and adult-onset primary Glaucoma, characterized by an autosomal-dominant/autosomal-recessive inheritance pattern with high penetrance.

Concerning the role of* MYOC* in PGC, possible interactions with* CYP1B1* and/or other unknown loci have been hypothesized to work as genetic modifiers to determine an earlier onset of the disease in patients harboring mutations in* MYOC* and* CYP1B1*, with respect to the patients negative for* MYOC* or* CYP1B1* mutations [25, 26, 36].

Another putative PCG-associated gene is* FOXC1*, a protein expressed in periocular mesenchyme cells, producing ocular drainage structures as iris, cornea, and trabecular meshwork. Interestingly, a deletion in* FOXC1* has been detected in PCG and other ocular and nonocular defects, highlighting a possible involvement of* FOXC1* in the pathogenetic pathway of the disease [25, 26].

Mutations in* BMP4* gene (14q22-q23) have been identified in patients affected by PCG and other disease phenotypes.* BMP4* codes for the bone morphogenetic protein 4 and it is expressed in different tissues, among which is the ocular vesicle, and in the optic cup. However, further research is needed to clarify the role of* BMP4* in PCG pathogenesis [26].

## 5. The Genetic Counseling in the Management of PCG

As PCG is a severe disease characterized by a particular genetic base and familiarity, genetic counseling represents a useful tool for the disease management (clinical features of the disease, prevision of the disease progression, and medical complications) [37]. Genetic counseling is a communication activity oriented to help the patient and/or their family in receiving medical information concerning the genetic features associated with PCG. In particular, the genetic specialist has to explain the recurrence risk of PCG in relation to the presence of positive/negative familiarity and to the frequency of PCG in the general population. The construction of familiar pedigree may be of help for clarification of the genetic model of inheritance of the disease. In fact, although the majority of cases of PCG appear to be sporadic cases or to follow an autosomal-recessive inheritance pattern, in rare cases some families reported an autosomal-dominant inheritance model. After the clarification of the genetic base of the disease, the recurrence risk and familiarity for PCG have been assessed, and the specialist should suggest and give details about the possibility of performing a genetic test for the detection of PCG-causative mutations. In particular, as illustrated before, mutations in* CYP1B1*,* LTBP2*, and* MYOC* have been found to be causative of PCG among different worldwide populations [25]. However, the specialist has to put in evidence the variable expressivity and penetrance defects occurring in presence of causative mutations. In fact, it is important to clarify to the patient that the positivity for PCG-associated mutations does not always correspond to the development of the effective pathological phenotype and the disease may manifest at later age. In case of presence of PCG-associated mutations, the genetic specialist should suggest to the patient a constant follow-up of his own clinical conditions. This aspect is strongly important in case of siblings of children positive to* CYP1B1* mutations, where there is a 25% higher risk of PCG development because of the variable expressivity of the disease phenotype [25, 38].

To help understand the pros and cons of genetic tests, it is very important to perform the informative counseling prior and after the test (pre- and a posttest). In this context the explanation of biological aspects and technical approaches related to the genetic test may help in understanding limits of negative results, false-negatives, and the need of further investigations.

Unfortunately, some cases may result negative to the analysis of PCG-causative known mutations, so that undiscovered mutations in other unidentified genes may be possible (e.g., unknown genes mapped on* GLC3B* and* GLC3C *loci). Social and psychological implications of the lack of a responsible gene involve the impossibility to perform genetic tests for the prediction of the disease risk in families with a positive-PCG history. This aspect may appear as a “sword of Damocles” and may affect deeply the couple perspective in matter of family construction.

## 6. Conclusion

The visual loss is due to interruption/aberration/malformation of the normal functioning of the ocular structures. Early-onset ocular diseases usually have Mendelian inheritance, while common adult-onset disorders are inherited as complex traits. To date, several genetic and molecular studies have provided insights into the biological processes underlying many ophthalmic disorders. In this context, GWAS identified different genetic variants contributing to a number of common ocular complex disorders.

Glaucoma is a heterogeneous group of disorders causing irreversible blindness worldwide. As previously described, most forms of Glaucoma present a significant genetic component, characterized by the different causative or susceptibility genes identified through traditional (genetic linkage) or novel (GWAS) approaches. Among the different forms of Glaucoma, we focused our attention on the PCG, that is, the single most common childhood Glaucoma.

PCG is a rare disease affecting the structures of the anterior chamber of the eye anterior segment. Although rare, it is still the most common Glaucoma in infancy and causes a disproportionately high percentage of childhood blindness worldwide. It displays a strong heterogeneity in terms of disease phenotype and genotype. Genetically speaking, PCG presents a familiarity trend, with higher frequency in populations where consanguineous relationships are common (Saudi Arabia and Slovakia Gypsy populations). To date, several mutations in* CYP1B1*,* LTBP2*, and* MYOC* have been associated with the development of the disease, although the expressivity phenotype and the penetrance are variable. Given these data, pre- and posttest genetic counseling has an essential role for an adequate management of PCG, in terms of medical, social, and psychological impact of the disease.

It is important to remark that the eye has been at the forefront of translational gene therapy largely because of the availability of appropriate disease targets and its suitable anatomic features. These advantages have further fostered the research that culminated in the establishment of various clinical trials for the gene therapy of ocular diseases. In this perspective the identification of modifier genes should be encouraged as well as the analysis of the interaction between genes and environmental factors. The challenge for the future is to classify patients in relation to their genetic background, in order to provide personal treatment and management.
